# Experimental and theoretical studies for corrosion of molybdenum electrode using streptomycin drug in phosphoric acid medium

**DOI:** 10.1038/s41598-023-31886-0

**Published:** 2023-03-24

**Authors:** Shymaa S. Medany, Yahia H. Ahmad, Amany M. Fekry

**Affiliations:** Chemistry Department, Faculty of Science, Cairo University, Giza, 12613, Egypt

**Keywords:** Medical research, Chemistry, Materials science

## Abstract

Corrosion inhibition of molybdenum electrode in H_3_PO_4_ acid medium of different concentrations (3.0 to 13 M) has been investigated utilizing different electrochemical techniques. It was observed that the most corrosive concentration is 3.0 M orthophosphoric acid concentration. The effect of adding Cl^−^ to 3.0 M orthophosphoric acid in the concentration range of 0.1 to 1.0 M was also studied. This study showed that the most corrosive medium is 3.0 M containing 1.0 M chloride ion with the greatest rate of hydrogen production. In 3.0 M H_3_PO_4_ acid with 1.0 M of NaCl, the tested electrode's corrosion and hydrogen production may be successfully suppressed by adding Streptomycin of 10 mM concentration leading to high inhibition efficiency. The outcomes of the studies were confirmed by scanning electron microscopic examination. Additionally, a computational chemistry approach was used to investigate how streptomycin adsorbs and inhibits corrosion at the interface of metal surfaces, and the outcomes of the computational studies are in excellent accord with the experimental findings.

## Introduction

Recently, studying the deterioration of the metals and alloys is considered an essential process of different approaches. Whereas, metals and alloys are widely used as electrodes in various applications like fuel cells, sensors, solar cells, and batteries^1–5^. Addition of Molybdenum to Cr–Ni stainless steels affects their properties in many aspects^6–9^. In addition, it reduces the risk of passive film deterioration in chloride medium. Hence, it increases passive film thickness, which is, in turn, improves the alloy’s resistance for corrosion. For increasing the strength, hardenability, toughness, and wear/corrosion resistance of steels, cast iron, and superalloys, molybdenum (Mo), a refractory metal, is frequently used as an alloying element^10–13^. Besides, molybdenum is utilized significantly in numerous chemical applications. It is well-known that the metal's high corrosion protection is attributable to the formation of thin, constant, and unsolvable oxide film of MoO_2_, which protects the metal surface from further surface oxidation^14,15^. Therefore, utilization of inhibitors to reduce corrosion of metals in contact with aggressive conditions is crucial, especially compounds containing N, S, or O^16,17^. A family of green corrosion inhibitors known as antibacterial medications has been proven to slow down the corrosion of engineered materials in a variety of media^18^.

Streptomycin is a first-line medicine for the treatment of plague that is also frequently used to treat tuberculosis when combined with other medications^19^. With effective antibacterial action, an aminoglycoside antibiotic called streptomycin produced from Streptomyces griseus^20–23^ with inhibitory consequence on Gram-negative bacteria, it is a well-known vet medication for bacterial illnesses and livestock farming^24^. Therefore, streptomycin as an antibiotic drug, a bactericidal antibiotic, and containing nitrogen and oxygen heteroatoms may be utilized to prevent corrosion in a variety of metals and alloys^25^. Acid solutions are utilized in numerous engineering applications^26–31^. Qiang et al. studied corrosion inhibition of some metals e.g. steel, copper, …etc. in acidic media^27–30^. Qiang et al. investigated the inhibitory effect of Losartan Potassium (LP) drug as a corrosion inhibitor for Q235 steel in hydrochloric acid^29^. A strong adsorption of LP on Q235 surface was proved by low ΔE and high E_binding_ values^29^. Phosphoric acid is readily used in acid washing applications due to its superior chemical properties. Corrosion inhibitors can reduce and, in several situations, prevent metal corrosion in harsh media by reducing hydrogen formation^32–34^.

**Figure 1:**
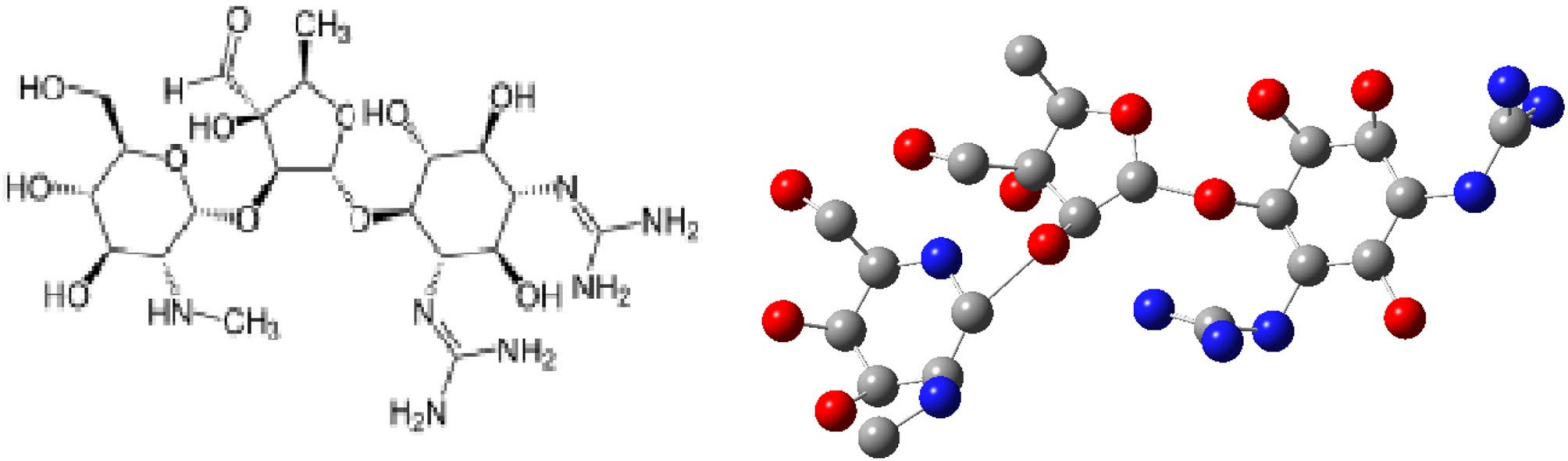
Schematic illustration of Streptomycin.

The chief purpose herein is to utilize surface examination procedures to investigate the electrochemical performance of molybdenum electrodes in various concentrations of an aerated H_3_PO_4_ solutions. Similarly, chloride ion with various concentrations (0.1–1.0 M) to 3.0 M H_3_PO_4_ solution was considered. Different levels of Streptomycin concentrations were investigated as an inhibitor for corrosion of molybdenum electrode in 3.0 M H_3_PO_4_ acid solution containing 1.0 M sodium chloride additive. The results of the experiment demonstrated that this substance considerably hinders the corrosion at 10^−2^ M concentrations of streptomycin. A direct relationship was found between the efficiency of corrosion inhibition and the concentration of the inhibitor in the presence of Cl^−^ ions in 3.0 M H_3_PO_4_ solution. Computational modeling was performed to validate the experimental results; suitability of Streptomycin as a corrosion inhibitor. This model can be used to extend the study to other concentrations that are not part of the current study. Figure 1 shows a schematic illustration of Streptomycin is as follows.

## Material and methods

A pure molybdenum electrode rod was prepared with a cross-sectional area of 1.0 cm^2^ in a cylindrical shape connected to a copper cable, covered with an adhesive epoxy resin coating made of Araldite, and injected in a glass cylinder. The electrode was refined by rubbing it with increasingly finer grades of emery paper (600–1600 grade), followed by a triple-distilled water rinse, bathed ultrasonically with acetone, and dehydrated in the air. The working electrode (WE) is a pure molybdenum electrode, platinum sheet and calomel electrode were used as auxiliary and reference electrodes, respectively. The three electrodes are inserted in a 25 ml cell containing the test solution.

The materials utilized are H_3_PO_4_, NaCl (Aldrich), and Streptomycin drug (antibiotic). The phosphoric acid solution is prepared in various concentrations (3.0 to 13 M), sodium chloride concentrations are 0.1–1.0 M and the inhibitor concentrations (Streptomycin) are 0.5–10 mM. All preparations used water that had been triple distilled. The electrochemical workstation IM6e from Zahner-electrik GmbH, Metechnik, Kronach, Germany, was employed to estimate electrochemical impedance spectroscopy (EIS) and polarization. The excitation AC voltage for the electrochemical impedance spectroscopy technique had a frequency range of 0.1 Hz to 100 kHz, and a peak-to-peak (p-p) amplitude of 10 mV. In comparison to a saturated calomel electrode, the sweeping rate was 30 mV min^−1^ across the potential operating voltage of −1000 to 0 mV. The intersecting of Tafel lines extension was implemented to derive the corrosion current density, abbreviated i_corr_. Using a computer least-squares analysis, the gradient of the points after E_corr_ by ± 50 mV was employed to derive Tafel constants. A scanning electron microscope (SEM) of JEOL-JEM-100s type with a 100× magnification was utilized for the surface investigation.

Streptomycin (C_21_H_39_N_7_O_12_) (molar mass = 581.574 g mol^−1^) is the first in class drug called aminoglycosides to be discovered^25,35^. It contains methoxy, amino, and hydroxyl groups.

## Results and discussion

### Potentiodynamic polarization measurements

**Figure 2:**
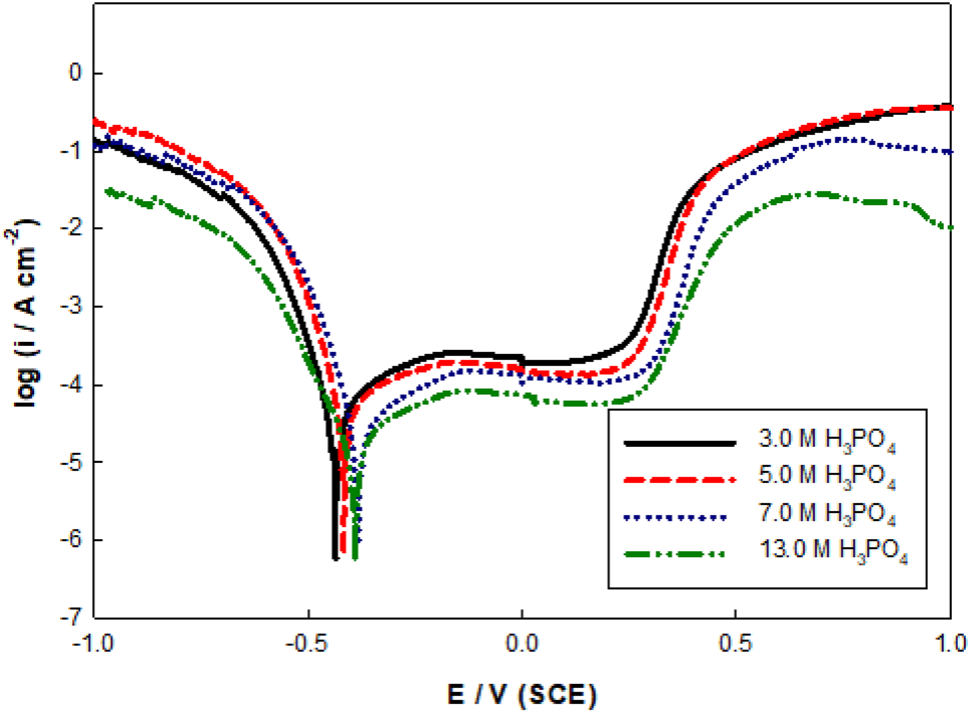
Potentiodynamic polarization curves in different concentrations of H_3_PO_4_ at 298 K.

Potentiodynamic polarization performance of the molybdenum electrode was evaluated in phosphoric acid solution (3.0 to 13 M). Figure 2 depicts a typical linear sweep potentiodynamic trace of the tested electrode in 3.0–13 M H_3_PO_4_. It was found that the E_corr_ motivated gradually towards positive direction as the acid concentration augmented and the i_corr_ was decreased owing to the development of different oxides of molybdenum (MoO_2_, MoO_3_, and Mo_2_O_5_) with increasing acidity. In comparison to neutral or basic media, the oxide coatings that develop on the Mo electrode are substantially highly stable in acidic ones. Also, hydrogen evolution was reduced under the same conditions. The data is given in Table 1. Following the determination of the altered molybdenum oxides' aqueous solution's thermodynamic durability, the associated equilibria could be evaluated. The passive film in acidic solutions consisted mostly of MoO_2_ in common with MoO_3_ and Mo(OH)_3_, which could be reduced using either Eq. (1) or Eq. (2):

1 $$2{\text{MoO}}_{3} + 2{\text{H}}^{ + } + {\text{e}} \to {\text{Mo}}_{2} {\text{O}}_{5} + {\text{H}}_{2} {\text{O}}$$

2 $${\text{MoO}}\left( {{\text{OH}}} \right)_{2} + {\text{H}}^{ + } + {\text{e}} \to {\text{MoOOH}} + {\text{H}}_{2} {\text{O}}$$

**Table 1:** Electrochemical corrosion parameters of Mo electrode as a function of phosphoric acid concentration at 298 K.

[H_3_PO_4_]/M	E_corr_/mV	i_corr_/μA cm^−2^	β_a_/mV/decade	β_c_/mV/decade
3.0	−440	69.2	154	−30.1
5.0	−420	58.9	123	−27.7
7.0	−383	23.4	112	−18.7
13	−382	17.4	124	−16.1

With a rise in acid concentration, an oxide layer developed on the electrode surface. The outcomes demonstrated that, in comparison to neutral and basic media, the oxide layer on Mo is somewhat highly stable in acidic medium. This behavior could be explained by the passive film's dominant element, MoO_2_, which comprises a negligible amount of MoO_3_ and Mo(OH)_3_. These Depending upon the solution's voltage and pH, these oxides may dissociate in aqueous solutions following the Eqs. (3, 4 and 5):

3 $${\text{MoO}}_{{2({\text{s}})}} + 2{\text{H}}_{2} {\text{O}}_{{({\text{l}})}} \to {\text{HMoO}}_{4}^{ - }{}_{{({\text{aq}})}} + 3H^{ + }{{({\text{aq}})}} + 2{\text{e}}$$

4 $${\text{MoO}}_{{2({\text{s}})}} + 2{\text{H}}_{2} {\text{O}}_{{({\text{l}})}} \to {\text{MoO}}_{4}^{2 - }{}_{{({\text{aq}})}} + 4{\text{H}}^{ + }_{{({\text{aq}})}} + 2{\text{e}}$$

5 $${\text{HMoO}}_{4}^{ - }{}_{{({\text{aq}})}} + {\text{H}}^{ + }_{{({\text{aq}})}} \to {\text{MoO}}_{3} + {\text{H}}_{2} {\text{O}}_{{({\text{l}})}}$$

In acidic media, these equilibrium systems will tend to stabilize the solid phase. Accordingly, the barrier film will be formed. Normally, in acidic solutions, ionic molybdate species are often reactive and have the tendency to polymerize to Mo_6_O_21_^6−^^36^. Therefore, the increase in acid concentration causes the large ionic species to polymerize, creating thick surface film. On the other hand, as the acid concentration rises, the rate of hydrogen evolution diminishes and also lowering the rate of corrosion.

**Figure 3:**
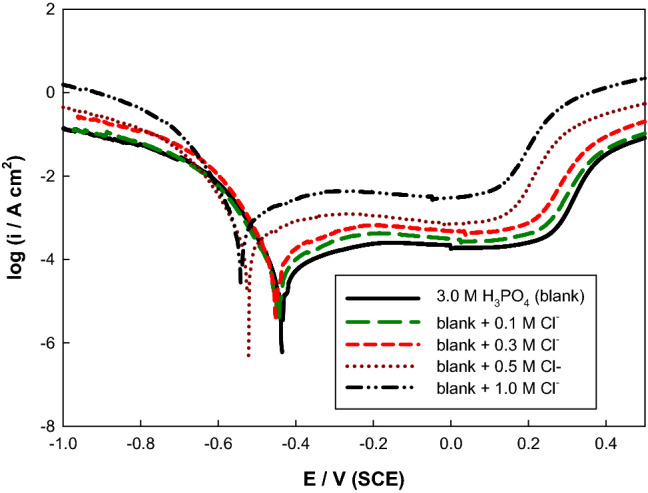
Potentiodynamic polarization curves of Mo in 3.0 M H_3_PO_4_ containing different concentrations of Cl^−^ at 298 K.

The main corroded phosphoric concentration is 3.0 M, so the polarization curves were developed for various concentrations of NaCl in 3.0 M H_3_PO_4_ acid solution. Figure 3 depicts the potentiodynamic curve for the utilized electrode in 3.0 M H_3_PO_4_ containing NaCl of different concentrations (0.1 to 1.0 M). The corrosion voltage moves noticeably to the active path, as shown in Table 2, causing depolarization of the anodic reaction by the anion, i.e., promoting the dissolution of the investigated electrode^17,37^.

**Table 2:** Electrochemical corrosion parameters of Mo electrode as a function of NaCl concentration in 3.0 M phosphoric acid at 298 K.

[Cl^−^]/M	E_corr_/mV	i_corr_/μA cm^−2^	β_a_/mV/decade	β_c_/mV/decade
0.1	−440	83.17	151	−30.1
0.3	−450	162.1	147	−35.2
0.5	−520	371.3	143	−30.8
1.0	−550	1023	145	−30.3

**Figure 4:**
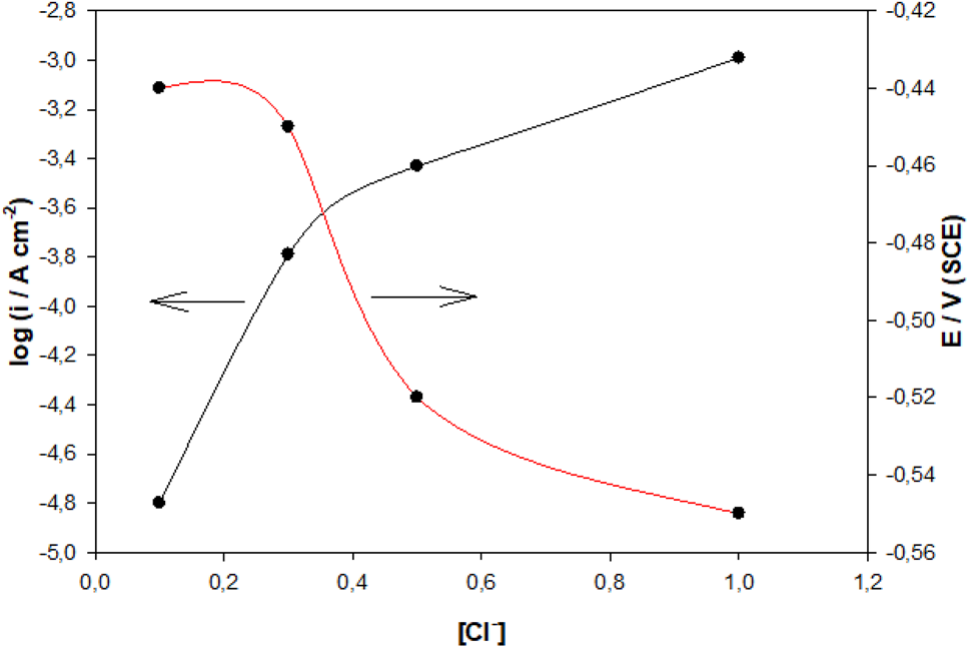
The variation of logi_corr_ and E_corr_ of Mo with Cl^−^ concentrations in 3.0 M H_3_PO_4_ solution at 298 K.

Figure 4 shows the connection between the i_corr_ and E_corr_ for NaCl concentration in 3.0 M H_3_PO_4_ at 298 K. i_corr_ value increases with the rise in Cl^−^ amount, which proposes that Cl^−^ ions contribute to form soluble oxochloro complexes leading to pitting nucleation at the active inclusion sites increasing the corrosion rate, or i_corr_. E_corr_ moves to more negative values, as demonstrated in Fig. 4 and Table 2.

**Figure 5:**
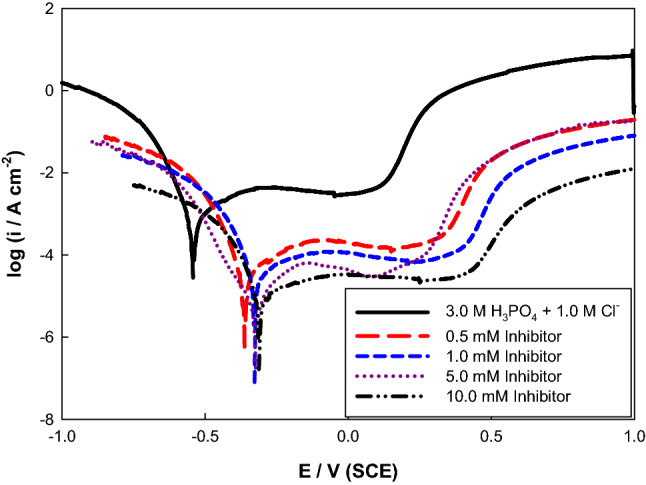
Potentiodynamic polarization curves of Mo in 3.0 M H_3_PO_4_ with 0.3 M Cl^−^ solutions containing different Streptomycin concentrations at 298 K.

By increasing anion concentration, it was observed that the hydrogen evolution and corrosion rate were greater in the acid-containing Cl^−^. The impact of streptomycin concentration (0.5–10 mM), as an inhibitor for the corrosion, was investigated in 3.0 M H_3_PO_4_ acid solution containing 1.0 M chloride (highly corrosive concentration or medium). The following equation was utilized to assess the inhibition efficiency (IE%) and the corrosion parameters listed in Table 3^38^:

6 $${\text{IE}}\% = 1 - \frac{{i_{inh} }}{{i_{corr} }} \times 100$$

where i_corr_ and i_inh_ are the uninhibited and inhibited corrosion current densities, respectively. It can be deduced that rising streptomycin concentration in 1.0 M Cl^−^ reduced i_corr_ and hydrogen formation at all concentrations. Hence, the inhibitor has led to the passivation of the studied electrode through adsorption and reduction of hydrogen formation. Because the inhibitor interacts with the metal surface through lone pairs of electrons on methoxy, hydroxyl, and/or amino groups that can form oxides, which effectively protect the metal surface, this can be credited to the accumulation of the inhibitor molecules by increasing their concentration on the Mo electrode. According to H^+^ ion or H_2_O molecule reduction, respectively, the main cathodic mechanism in Mo corrosion in acidic solutions is hydrogen evolution reaction^39^. Due to the surface-hindering effects of both adsorption and film formation, which decrease the attack area, the increase in inhibitor concentration enhanced the corrosion inhibition efficacy to 98.85% at 10.0 mM of inhibitor. The cathodic and anodic Tafel slopes changed normally as illustrated in Fig. 5, indicating the presence of hindering effect without altering the reaction mechanism.

**Table 3:** Electrochemical corrosion parameters of Mo electrode as a function of inhibitor concentration in 3.0 M phosphoric acid containing 1.0 M NaCl at 298 K.

[Inhibitor]/mM	E_corr_/mV	i_corr_/μA cm^−2^	β_a_/mV/decade	β_c_/mV/decade	IE%
0.5	−360.5	45.71	87.9	−31.6	95.53
1.0	−315.1	43.65	86.3	−34.3	95.73
5.0	−306.6	19.59	80.6	−34.6	98.09
10	−298.4	11.74	76.5	−40.1	98.85

The Tafel behavior of the Butler-Volmer Model was estimated as follows^40–42^:

7 $$\eta=\frac{2.303 RT }{(1-\propto)nF } {\mathrm{Log}}\, {j}_{o}+\frac{2.303 RT }{(1-\propto)nF} {\mathrm{Log}}\, j$$

The uppermost IE (%) can be attributed to the -OCH_3_, NH_2_, OH, or C=O groups and/or π-electrons of the double bond^43^. Hydrogen formation is of great significance for hydrogenation reactions in the acid medium as phosphoric acid. Subsequent mechanisms may be suggested for hydrogen evolution reaction on electrodes in acidic solutions^44,45^:

1. a principal discharge (Volmer reaction)

8 $${\text{M}} + {\text{H}}_{3} {\text{O}}^{ + } + {\text{e}} \leftrightarrow {\text{MH}}_{{{\text{ad}}}} + {\text{H}}_{2} {\text{O}}$$

2. a desorption step (Heyrowsky reaction)

9 $${\text{MH}}_{{{\text{ad}}}} + {\text{H}}_{3} {\text{O}}^{ + } + {\text{e}} \to {\text{M}} + {\text{H}}_{2} + {\text{H}}_{2} {\text{O}}$$

3. a combination step (Tafel reaction)

10 $${\text{MH}}_{{{\text{ad}}}} + {\text{MH}}_{{{\text{ad}}}} \to 2{\text{M}} + {\text{H}}_{2}$$

First, hydronium ion is discharged^46^. No reaction can happen alone, however, associated with anotherVolmer response must be slow if Tafel and/or Heyrowsky reaction are both rapid. A sluggish step followed by a quick step. Hence, the inhibitor's existence may prevent MH_ads_ formation or the electron move to hydronium ion and suppress both reactions (7 & 8, respectively).

In destructive environments, the atomic hydrogen (MH_ads_) will resyndicate, producing molecular hydrogen collected on the surface as a second step of the HER.

### EIS measurements

**Figure 6:**
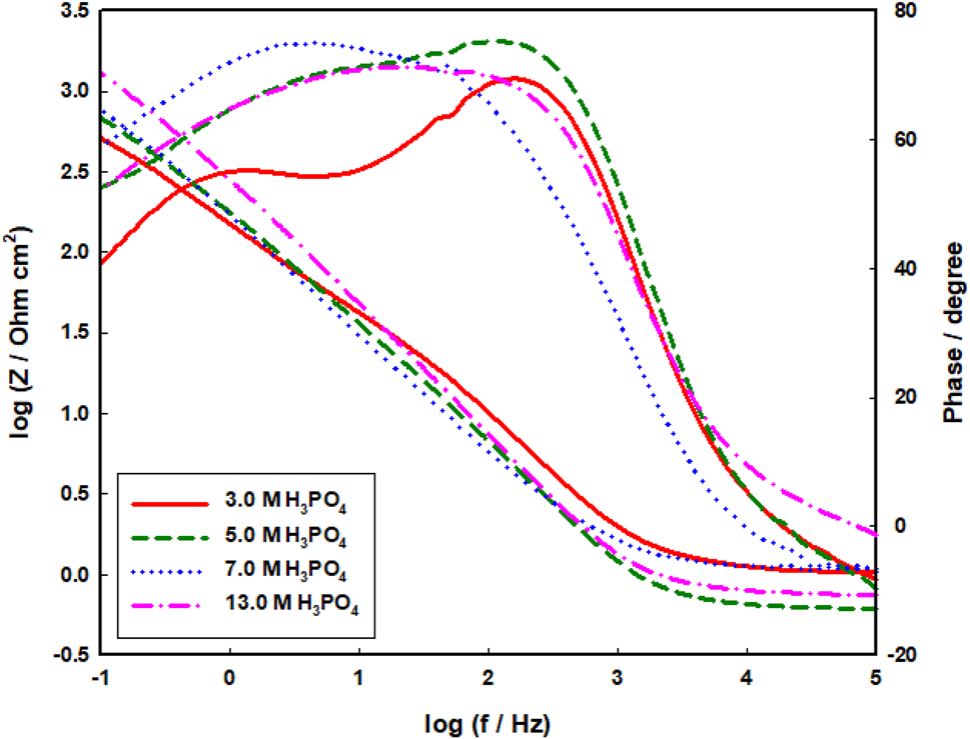
Bode plots of Mo in different concentrations of H_3_PO_4_ at 298 K.

**Figure 7:**
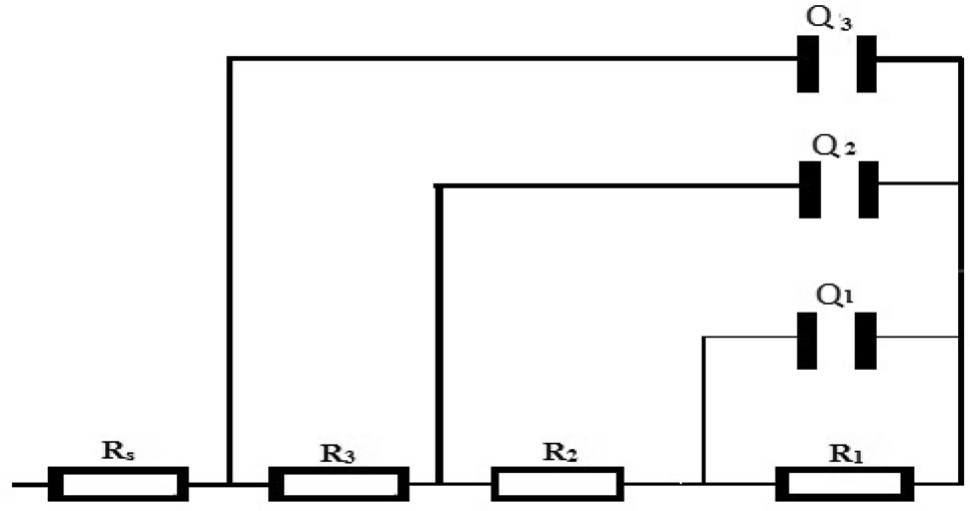
An equivalent circuit model used to fit the impedance data of Mo.

Figure 6 displays the EIS data for Mo electrodes in phosphoric acid of different concentrations (3.0–13 M). Bode plots demonstrated a wide maximum phase diagram, representing the existence of three-time constants^47–49^. Fitting of the spectra has been done by means of a three-time constant model in which three parallel CPEs (Q_1_, Q_2,_ and Q_3_) were utilized (Fig. 7). The interfacial impedance (Z) is defined by^50,51^:

11 $$Z\left( \omega \right) = \sum\limits_{L = 1}^{L = 3} {\frac{{R_{L} }}{{1 + (j\omega )^{x} R_{L} Q_{L} }} + R_{s} }$$

where x = 1 resembles a perfect capacitor, then the fitting information displayed that x values < 1. Thus, at ω = 1, the total reciprocal capacitance is:

12 $$\frac{I}{{C_{T} }} = \frac{I}{{C_{1} }} + \frac{I}{{C_{2} }} + \frac{I}{{C_{3} }}$$

The reciprocal capacitance enlarges linearly with growing acid concentration. The simulated and the experimental outcomes indicating a well-suited model with a 3% fit error. The comparable circuit parameters are introduced in Table 4. The data indicate that R_T_ values enlarged with growing acid concentration.

**Table 4:** Impedance parameters of Mo electrode as a function of phosphoric acid concentration at 298 K.

[H_3_PO_4_]/M	R_1_/kΩ cm^2^	Q_1_/μF cm^−2^	α_1_	R_2_/Ω cm^2^	Q_2_/μF cm^−2^	α_2_	R_3_/Ω cm^2^	Q_3_/μF cm^−2^	α_3_	R_s_/Ω
3.0	10.2	11.2	0.80	25.0	19.1	0.64	0.31	22.1	0.86	1.0
5.0	13.1	10.1	0.84	160	15.9	0.67	0.70	20.6	0.87	0.7
7.0	14.4	8.35	0.86	170	13.3	0.67	0.75	20.5	0.85	1.1
13	19.7	6.50	0.90	192	10.9	0.69	0.77	17.3	0.85	0.8

The reciprocal capacitance enlarges linearly with growing acid concentration. The simulated and the experimental outcomes indicating a well-suited model with a 3% fit error. The comparable circuit parameters are introduced in Table 4. The data indicate that R_T_ values enlarged with growing acid concentration.

**Figure 8:**
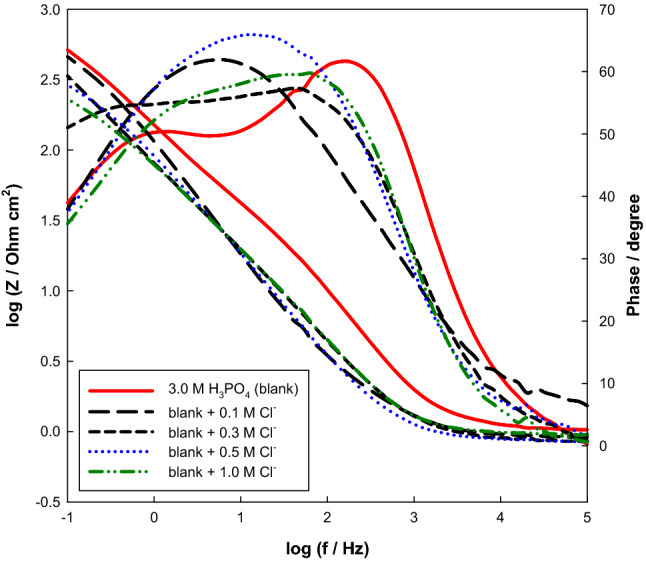
Bode plots of Mo in 3.0 M H_3_PO_4_ containing different concentrations of Cl^−^ at 298 K.

As shown in Fig. 8, the Bode plots for various NaCl concentrations in 3.0 M H_3_PO_4_ acid solution are fitted with a similar model given in Fig. 7 and the results are in Table 5. The relative thickness (1/C_T_) and the total resistance (R_T_) of the film decrease with increasing anion concentration.

**Table 5:** Impedance parameters of Mo electrode as a function of NaCl concentration in 3.0 M phosphoric acid at 298 K.

[Cl^−^]/M	R_1_/kΩ cm^2^	Q_1_/μF cm^−2^	α_1_	R_2_/Ω cm^2^	Q_2_/μF cm^−2^	α_2_	R_3_/Ω cm^2^	Q_3_/μF cm^−2^	α_3_	R_s_/Ω
0.1	9.35	14.7	0.76	19.7	21.3	0.78	0.29	29.3	0.79	0.96
0.3	7.01	16.5	0.74	18.6	23.5	0.76	0.27	31.2	0.78	0.94
0.5	6.67	16.9	0.75	18.3	25.9	0.71	0.26	39.6	0.78	0.92
1.0	5.63	18.0	0.73	17.5	26.3	0.70	0.21	50.1	0.72	0.87

**Figure 9:**
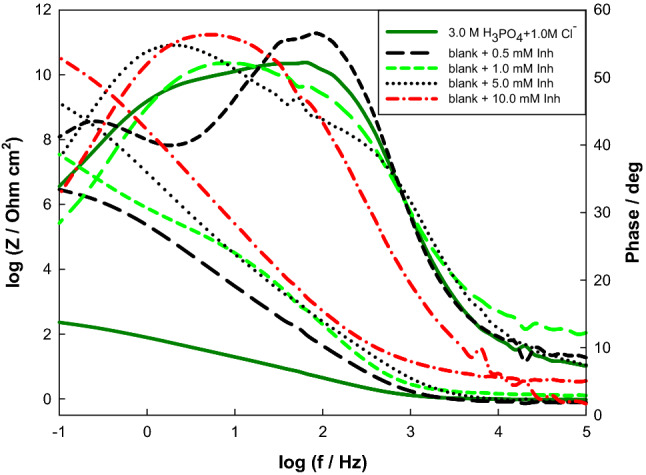
Bode plots of Mo in 3.0 M H_3_PO_4_ with 0.3 M Cl^−^ solutions containing different Streptomycin concentrations at 298 K.

Inhibition of corrosion happens by adding the inhibitor to the highest corrosive medium (3.0 M H_3_PO_4_ containing 1.0 M chloride ion), with concentrations (0.5–10 mM) as displayed in Fig. 9. The data were best fitted with the model presented in Fig. 7 and fitted factors are provided in Table 6. Given that the passive oxide film may be compared to a dielectric plate capacitor, the equation below relates the passive film thickness (*d*) in cm to the capacitance (*C*)^52–54^:

13 $${\text{d}} = \varepsilon_{o} \varepsilon_{r} {\text{A}}/{\text{C}}$$

where ε_o_ is the vacuum permittivity (0.885 × 10^−11^ Fcm^−1^), ε_r_ is the comparative dielectric constant of the film, and *A* is the electrode surface area in cm^2^. Although the genuine estimation of εr inside the film is hard to assess, a variation of C can express a difference in the film thickness. The 1/C and thickness are related directly to each other. Consequently, as the concentration of the inhibitor grows, extra inhibitor units will be accumulated on the surface via the active centers (heteroatoms or double bonds) in streptomycin, promoting an increment in film thickness and diminishing in H_2_ advancement.

**Table 6:** Impedance parameters of Mo electrode as a function of inhibitor concentration in 3.0 M phosphoric acid containing 1.0 M NaCl at 298 K.

[Inhibitor]/mM	R_1_/MΩ cm^2^	Q_1_/μF cm^−2^	α_1_	R_2_/kΩ cm^2^	Q_2_/μF cm^−2^	α_2_	R_3_/Ω cm^2^	Q_3_/μF cm^−2^	α_3_	R_s_/Ω	IE%
0.5	0.2	10.3	0.79	5.5	13.5	0.70	6.5	18.1	0.62	6.15	97.3
1.0	0.3	9.8	0.75	6.1	12.8	0.72	9.7	17.5	0.67	6.36	98.2
5.0	0.6	7.1	0.76	6.5	12.5	0.73	11	16.8	0.68	5.46	99.1
10	1.5	3.5	0.80	9.0	11.9	0.75	16	16.1	0.69	9.62	99.6

Chloride ions can form negative charges in the interface region because of specific adsorption, and then, the streptomycin compound is protonated in the acid solution. This leads to strong adsorption of protonated drug and chloride ions, thus preventing chloride ions to attack Mo electrode surface which has positive charges in the acidic medium. The concentrations of Cl and protonated medicines are then substantially higher than those in bulk near to the interface. Due to the repulsion force between the protonated inhibitor and the positively charged surface, the protonated inhibitor medication does not directly attack the positively charged surface. It can be attached to the molybdenum surface by electrostatic contact between Cl^−^ and protonated inhibitor. The inhibitor adsorbed on the metal through different polar groups (–OCH_3_, NH_2_, OH, or C=O) by a coordination bond. The efficiency of inhibition reaches 99.6% at 10.0 mM of inhibitor concentration which is in respectable promise with that of polarization outcomes.

**Figure 10:**
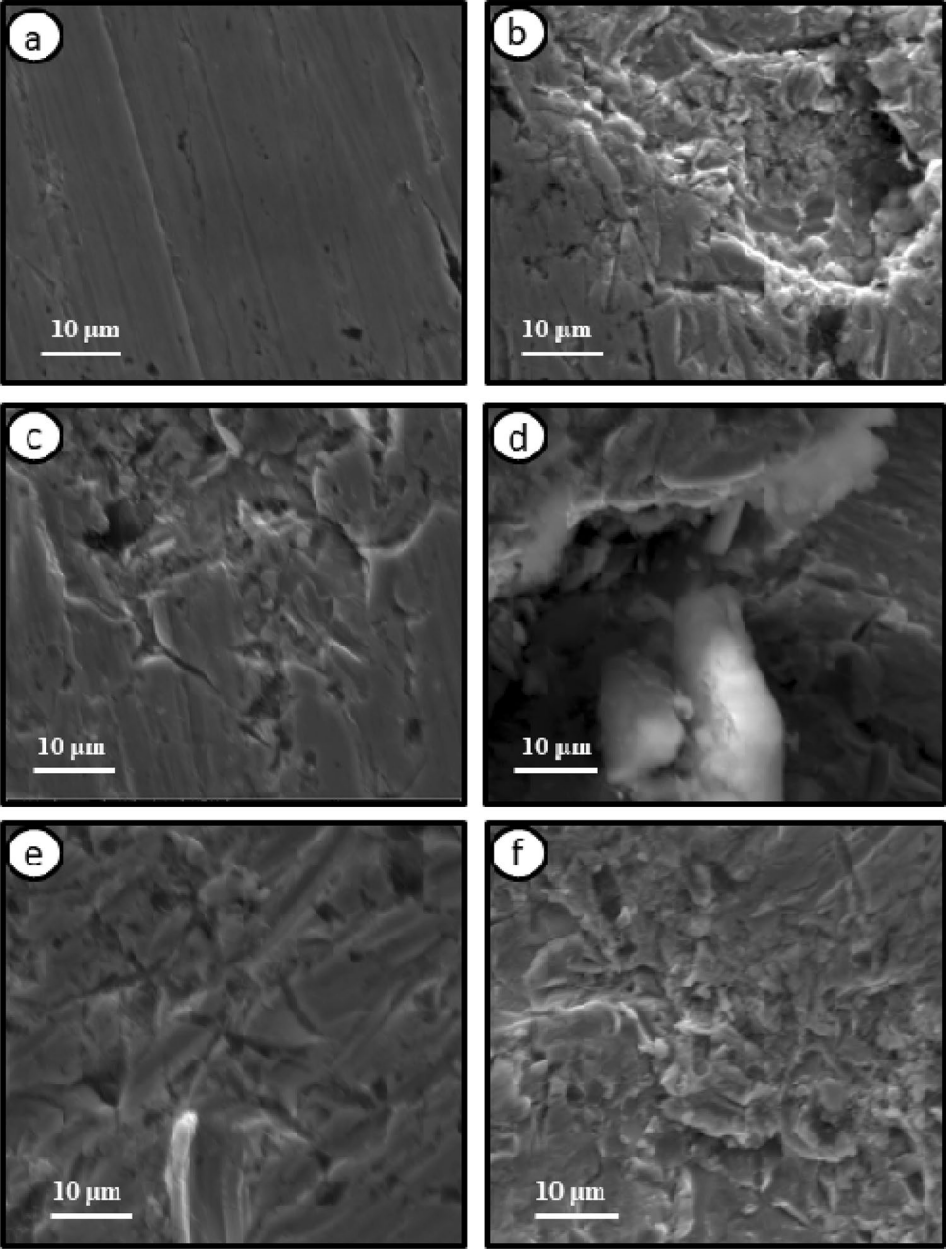
SEM images of Mo surface (**a**) mechanically polished and after 2 h of immersion in (**b**) 3.0 M H_3_PO_4_ solution, (**c**) 13 M H_3_PO_4_, (**d**) 3.0 M H_3_PO_4_ solution comprising 1.0 M Cl^−^, (**e**) 3.0 M H_3_PO_4_ solution comprising 1.0 M Cl^−^ and 0.5 mM Streptomycin and (**f**) 3.0 M H_3_PO_4_ solution comprising 1.0 M Cl^−^ and 10 mM Streptomycin.

The outcomes were verified by surface examination. Figure 10 signifies an example for the SEM image for the tested electrode in the air (Fig. 10a), which is a smooth sample, 3.0 M H_3_PO_4_ (Fig. 10b) is corroded surface with large pores, 13 M H_3_PO_4_ (Fig. 10c) is much more smooth surface containing some scratches, 3.0 M H_3_PO_4_ with 1.0 M NaCl (Fig. 10d) is corroded surface with precipitates of salts, 3.0 M H_3_PO_4_ with 1.0 M NaCl containing 0.5 mM streptomycin (Fig. 10e) is so smooth surface without large pores and with increasing inhibitor concentration till 10 mM streptomycin (Fig. 10f), the surface becomes more smoother.

### Computational calculations

Gaussian quantum chemical calculations view 5.08 program is a significant matter to predict the inhibitor's molecular mechanism for adsorption on the tested alloy surface. The goal is to explore the applicability of quantum–mechanical calculations to expect the inhibition efficiency of Streptomycin. The computed quantum chemical parameters for Streptomycin are 155 alpha electrons and 154 beta electrons that can be included in the coordinate bond to be adsorbed well on the surface. This ensures its well absorbability. The Molecular volume (MV) determined as 460 cm^3^/mol and molecular surface area is 557 cm^2^. This means that the area is large enough for the inhibitor to cover the metal surface. Hence, it provides an extensively high resistance effect on surface of the metal, with noticeable inhibition efficiency growth^55^. The nuclear-nuclear repulsion E_NN_ describes the electrostatic repulsion between the nuclei and is found to be 5458.69 Hartees = 148,538.5096 eV. It is also so high which confirmed the well adsorption ability of the inhibitor. Thus, its E_NN_, large surface molecular area and large number of alpha and beta electrons included ensure its high absorbability. Also, from calculations, ExpMin = 3.60D-02, ExpMax = 8.59D+03, ExpMxC = 1.30D+03. This means that it acts well as an electron donor compound to be adsorbed well on the alloy surface through these electrons with a coordinate bond^56^.

## Conclusions

As a result of potentiodynamic polarization and EIS estimations, using surface examination and quantum chemical calculations, the following points were concluded:

- The values of i_corr_ declined with the rise of the molar concentrations of phosphoric acid.
- The values of i_corr_ increased with the rise in anion concentration and decreased with the rising in inhibitor concerntration.
- Quantitative research that is based on the CPE idea provides a greater level of agreement between experimental findings and theoretical data, showing the applicability of the suggested model (two-time constants) for elucidating real-world data.
- The total resistance R_T_ values increase with rising inhibitor concentration in 3.0 M phosphoric acid containing 1.0 M NaCl at 298 K.

## Data Availability

The datasets used and/or analyzed during the current study available from the corresponding author on reasonable request.
